# Exogenous fibroblast growth factor 1 ameliorates diabetes-induced cognitive decline via coordinately regulating PI3K/AKT signaling and PERK signaling

**DOI:** 10.1186/s12964-020-00588-9

**Published:** 2020-05-27

**Authors:** Yanqing Wu, Chengbiao Wu, Libing Ye, Beini Wang, Yuan Yuan, Yaqian Liu, Peipei Zheng, Jun Xiong, Yiyang Li, Ting Jiang, Xiaokun Li, Jian Xiao

**Affiliations:** 1 The Institute of Life Sciences, Engineering Laboratory of Zhejiang province for pharmaceutical development of growth factors, Biomedical Collaborative Innovation Center of Wenzhou, Wenzhou University, Wenzhou, 325035 China; 2 Molecular Pharmacology Research Center, School of Pharmaceutical Science, Wenzhou Medical University, Wenzhou, 325035 Zhejiang China; 3 Clinical Research Center, Affiate Xiangshang Hospital, Wenzhou Medical University, Wenzhou, Zhejiang China

**Keywords:** Diabetes-induced cognitive decline (DICD), Fibroblast growth factor 1 (FGF1), Hippocampus, Neuronal apoptosis, Protein kinase RNA-like ER kinase (PERK), cAMP-response element binding protein (CREB)

## Abstract

**Background:**

Diabetes induces central nervous system damage, leading to cognitive decline. Fibroblast growth factor 1 (FGF1) has dual function of neuroprotection and normalizing hyperglycemia. To date, the precise mechanisms and potential treating strategies of FGF1 for diabetes-induced cognitive decline (DICD) hasn’t been fully elucidated.

**Methods:**

In this study, db/db mice were used as DICD animal model. We found that diabetes remarkably suppressed FGF1 expression in hippocampus. Thus, exogenous FGF1 had been treated for db/db mice and SH-SY5Y cells.

**Results:**

FGF1 significantly ameliorates DICD with better spatial learning and memory function. Moreover, FGF1 blocked diabetes-induced morphological structure change, neuronal apoptosis and Aβ_1–42_ deposition and synaptic dysfunction in hippocampus. But normalizing glucose may not the only contributed factor for FGF1 treating DICD with evidencing that metformin-treated db/db mice has a inferior cognitive function than that in FGF1 group. Current mechanistic study had found that diabetes inhibits cAMP-response element binding protein (CREB) activity and subsequently suppresses brain derived neurotrophic factor (BDNF) level via coordinately regulating PERK signaling and PI3K/AKT signaling in hippocampus, which were reversed by FGF1.

**Conclusion:**

We conclude that FGF1 exerts its neuroprotective role and normalizing hyperglycemia effect, consequently ameliorates DICD, implying FGF1 holds a great promise to develop a new treatment for DICD.

**Video abstract**

## Background

Diabetes could induce central nervous system (CNS) damage, leading to neurophysiological and structural changes, and consequently cognitive decline. Diabetes-induced cognitive decline (DICD) manifests many characteristics of chronic encephalopathy, such as decline of learning and memory ability, impairment of language, understanding and judgment [1], which seriously affects the living quality of patient. With the dramatic increases of diabetes, it is of great significance to further explore the molecular mechanisms and potential therapeutic strategies for DICD.

cAMP-response element binding protein (CREB), a family of leucine zipper transcription factors, is widely expressed in many tissues, including the brain. Its transcriptional activity is co-regulated by phosphorylation levels of serine residues at sites of 133 and 129. Phosphorylation of CREB at S133 is critical for CREB recruitment of coactivator CREB binding protein (CBP) to form active transcription complexes, and it is regulated by cAMP-protein kinase A (PKA), MAP kinase signaling, and protein kinase B (AKT) [2–4]. CREB activity regulates the expressions of memory consolidation and long-term potentiation (LTP)-related genes, including c-fos, activity-regulated cytoskeletal associated protein (Arc) and brain derived neurotrophic factor (BDNF) [5]. As a neurotrophic factor, BDNF is crucial for the growth and normal function of nerve cells. CREB activity and BDNF expression are remarkably suppressed in Alzheimer disease (AD) patients and AD animal models [6], and it is associated with cognitive decline [7]. Additionally, *CREB* gene knockout affected spatial memory formation of mice under fear condition [8, 9]. We speculated that CREB maybe also an important molecular target during pathogenesis of DICD.

Endoplasmic reticulum (ER) stress mainly occurs in axon, dendrite and dendritic spines in neuron, and involved in the regulation of neurodegenerative disease, especially protein kinase RNA-like ER kinase (PERK) signaling pathway that is overactivated in AD patients [10–14]. Mechanism studies have shown that phosphorylated PERK activates eIF2α and subsequently triggers cell apoptosis. Moreover, PERK-eIF2α signaling not only regulates the transition from short-term to long-term memory, but also affects synaptic plasticity [13, 15]. Independent of eIF2α, PERK signaling also suppresses BDNF expression through phosphorylating CREB at S129 and PSD95, and then affects the stability of dendritic spines and mediates memory decline after traumatic brain injury (TBI) [14, 16]. Thus, we speculated that PERK signaling may participate in the regulation of CREB activity during DICD development.

Phosphoinositide 3 kinase/protein kinase B (PI3K/AKT) signaling pathway, a classical signaling pathway in mammals, is involved in the regulatory process of cerebrovascular diseases, neurodegenerative diseases, and demyelination diseases. Increasing evidences have shown that PI3K/AKT pathway is closely related to synaptic plasticity, learning and memory [17], and inhibited during AD occurrence and development [18]. More importantly, AKT is one of the major kinases that regulates CREB activity. AKT suppression inhibits the p-CREB (S133) level, reduces CREB activity, and then participates in the regulation of neuron survival and synaptic function in AD and Parkinson’s disease (PD) development [3, 19]. Therefore, we speculated that cooperating with PERK pathway, PI3K/AKT signaling pathway maybe also involved in the regulation of CREB activity during DICD.

Fibroblast growth factor 1 (FGF1), an important member of fibroblast growth factors (FGFs), regulates the growth and proliferation of various types of cells by binding with heparan sulfate protein receptor. As a neurotrophic factor, FGF1 promotes the survival and regeneration of injured nerve [20, 21]. More importantly, its safety and efficacy have been confirmed in clinical trials [22]. Additionally, as an insulin sensitization, FGF1 effectively normalizes the hyperglycemia of type 2 diabetes without adverse effects [23]. It has been reported that FGF1 alleviates neuronal apoptosis and consequently ameliorates PD disease by promoting PI3K/AKT signaling and inhibiting elevated ER stress [24]. We supposed that FGF1 may exert its dual role of anti-diabetics and neuroprotection, and participate in the regulation of DICD development.

In this study, db/db mice were used as DICD animal model, and to investigate the role of PI3K/AKT signaling and PERK signaling for CREB activity and neuronal apoptosis during DICD development. To date, the role of FGF1 on development of DICD has not been well described. Here, we have further explored whether FGF1 administration can block PI3K/AKT signaling and PERK signaling, and ameliorate DICD development.

## Materials and methods

### Animal and experimental design

**Fig. 1:**
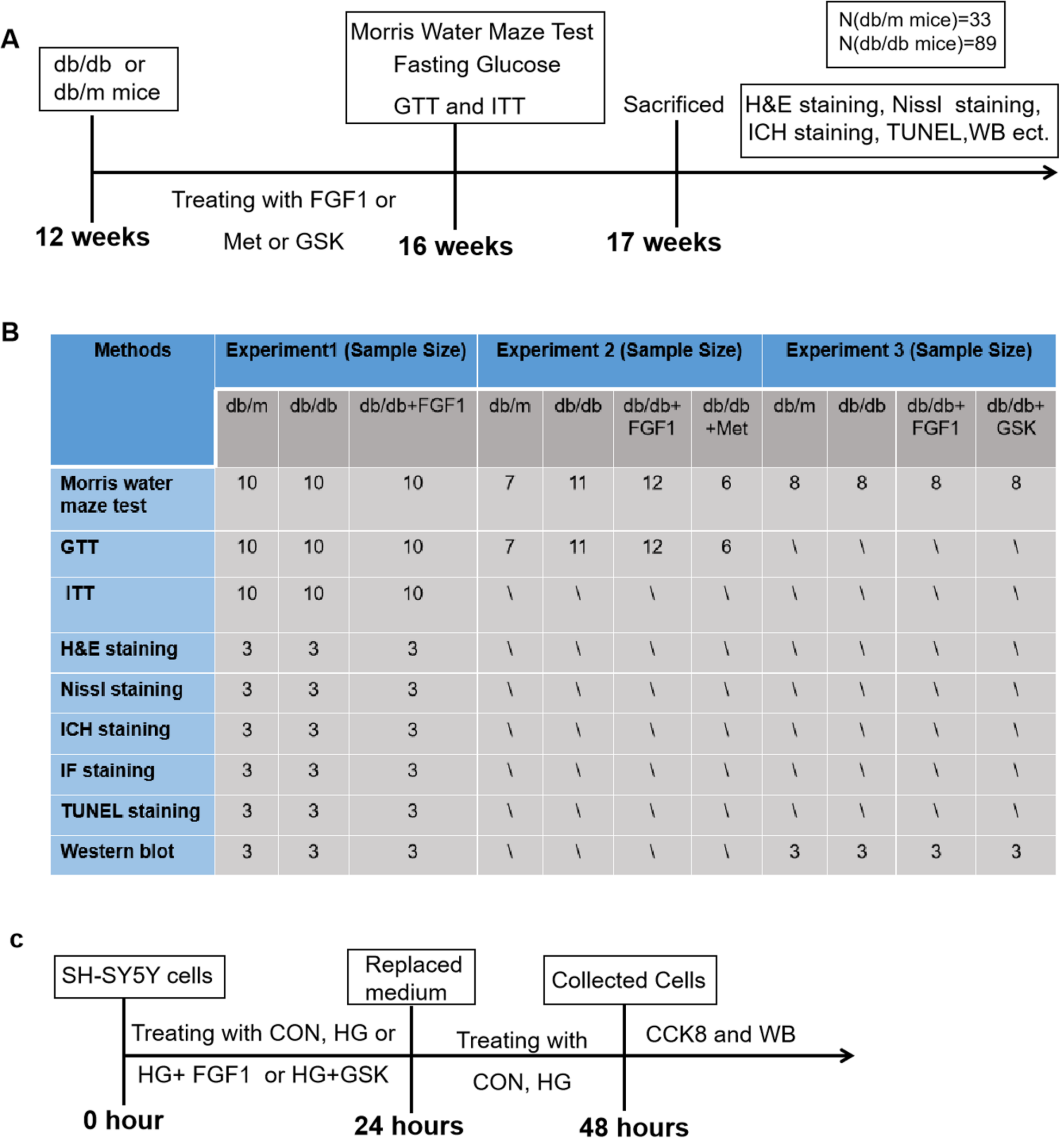
The time duration of animal study and SH-SY5Y cells study. **a** The time duration of animal study; **b** The sample pet group in every experiment; **c** The time duration of SH-SY5Y cells study

Twelve-week old male db/db (C57BLKS/J-leprdb/leprdb) mice and their non-diabetic db/m litter mates were purchased from the Model Animal Research Center of Nanjing University (Nanjing, China). All experimental procedures were performed in accordance with National Institutes of Health guide for the care and use of Laboratory animals. The animals were maintained under a 14-h light/10-h dark condition. After arrived, the animals were acclimatized to animal house before use. The db/db mice were divided into two groups, and intraperitoneally (i.p.) injected either with FGF1 (0.5 mg/kg body weight) [25] or physiologic saline every other day for 4 weeks (Fig. 1a and b). To verify whether normalizing hyperglycemia is the main contribute factor for FGF1 treating DICD, 200 mg/kg metformin [26, 27], a classical anti-diabetic drug, was administrated to db/db mice as positive control group for 4 weeks. To further detected the role of PERK signaling during FGF1 treating for DICD 50 mg/kg GSK2656157 (GSK) [14], a PERK activity inhibitor, was administrated for db/db mice for 4 weeks to inhibit PERK activity. After 4 weeks, glucose tolerance test (GTT) and insulin tolerance test (ITT) were tested, and then the mice were performed Morris water maze test. Then, they were anesthetized with 10% chloral hydrate (3.5 mL/kg) and perfused via cardiac puncture initially with 0.9% saline solution. For immunofluorescence and TUNEL assay, the brains were rapidly detached and embedded in O.C.T compound (Changzhou, Jiangsu, China) for frozen sectioning. For hematoxylin & eosin (H&E) staining and immunohistochemistry, animals were perfused with 4% paraformaldehyde (PFA) in 0.1 M PBS following the saline solution perfusion. Then, the brains were rapidly detached and post-fixed by immersion in 4% PFA for 24 h. For western blotting analysis, the hippocampus was separated and rapidly stored at − 80 °C.

### Blood glucose measurement, glucose tolerance test (GTT) and insulin tolerance test (ITT)

Blood glucose level was measured using a handheld glucometer with appropriate test strips (FreeStyle Lite). For GTT, the mice were fasted overnight, and then intraperitoneally injected with glucose at a dose of 2 g/kg body weight. Blood glucose levels were measured prior to injection, and at 15, 30, 60, 90, and 120 min time point after glucose injection. The trapezoidal rule was used to determine the area under the curve (AUC) of blood glucose during GTT. For ITT, the mice were fasted for 5 h, and then intraperitoneally injected with insulin at a dose of 0.75 U/kg body weight. Blood glucose levels were measured prior to injection, and at 15, 30, 60, 90, and 120 min time point after injection.

### Morris water maze test

The test [28] was performed in a circular pool with a diameter of 120 cm and a height of 40 cm (Jiliang, Shanghai, China). It was filled with opaque water colored with milk powder and maintained at a temperature of 26 ± 1 °C. Using a hidden circular platform, the training was carried out with six blocks that consisted of three 60-s trials separated by 20 min inter-block intervals. During the training, the platform remained in the same location relative to the distal cues in the room. For each trial, mice were placed in the water at different start locations (E, S, W, and N) that were equally spaced from each other and were offset from the goal location by 45°. One hour following the sixth block, the hidden platform was removed, and the mice were scored during a 60 s probe trial. They were scored for latency to reach the goal and for memory recall, which was determined by crossing over the previous platform location. Another probe trial was performed 24 h after training to assess memory consolidation and memory retrieval.

### H&E staining, Nissl staining and immunohistochemistry staining

For H&E staining, the 5 μm sections were dewaxed and hydrated, then stained with hematoxylin and eosin solutions, and observed under light microscope. For Nissl staining, tissue sections were stained with cresol violet and Nissl differentiation solutions according to the instructions (Beyotime), and observed under light microscope. For Immunohistochemistry staining, the sections were incubated with 3% H_2_O_2_ for 15 min, and then in blocking solution for 45 min. Subsequently, the sections were incubated at 4 °C overnight with the following primary antibody: Aβ_1–42_ (1:400, Abcam). After washing in PBS for 3 times, the sections were incubated with horseradish peroxidase-conjugated secondary antibodies for 4 h at 37 °C. Then, the sections were reacted with 3, 3-diaminobenzidine (DAB), and imaged using a Nikon ECLPSE 80i (Nikon, Tokyo, Japan).

### Immunofluorescence staining

The 5 μm frozen sections were fixed by precooled acetone for 15 min and then washed with PBS for 3 times. Then, the sections were respectively incubated with 5% bovine serum albumin (BSA) in 37 °C oven for 0.5 h and following primary antibodies in 4 °C overnight: FGF1(1:200, abcam), p-Tau (1:200, abcam), synaptophysin (1:150, abcam), PSD95(1:150, abcam), p-PERK (1:200, cell signaling technology), BDNF (1:150, abcam), and MAP 2(1:2000, abcam). After triple washing in PBS at room temperature, the sections were once again incubated with Alexa Fluor 647 (1:1000, Abcam) as secondary antibody for 4 h. Fluorescence images were captured using a Nikon ECLPSE 80i (Nikon, Tokyo, Japan).

### Western blotting analysis

For protein extraction, the hippocampus was homogenized in lysis buffer containing protease inhibitor cocktail (10 μl/ml, GE Healthcare Biosciences, PA, USA). Then, the complex was centrifuged at 12,000 rpm, and the supernatant was obtained for the protein assay. SH-SY5Y cells were lysed in cell lysis buffer with protease and phosphatase inhibitors. The extracted protein was quantified with BCA reagents. The protein was separated on a 8% or 12% gel, and transferred onto a PVDF membrane (Bio-Rad, Hercules, CA, USA). The membrane was blocked with 5% milk in TBS for 0.5 h. and incubated with primary antibodies in TBS for 2 h at room temperature or overnight at 4 °C. After washed with TBST (TBS with 0.05% tween 20) for 3 times, the membrane was treated with horseradish peroxidase-conjugated secondary antibodies (1:3000) for 2 h at room temperature. Signals were visualized by ChemiDocXRS+Imaging System (Bio-Rad). All experiments were repeated in triplicate with independently prepared tissue. The densitometric value of band was obtained by Image J software and subjected to statistical analysis.

### TUNEL staining

TUNEL staining was performed using the ApopTag Fluorescein Direct In Situ Apoptosis Detection Kit (Roche, Basel, Switzerland). According to the standard protocol, the frozen sections were fixed with precooled acetone for 15 min and washed with PBS for 3 times. Then, these sections were incubated with 20 μg/ml proteinase K working solution for 15 min at 37 °C. The slides were then rinsed with PBS for 3 times, and incubated with TUNEL reaction mixture for 1 h at 37 °C. After rinsing with PBS for 3 times, the sections were stained with 4′, 6-diamidino-2-pheny-lindole (DAPI, Beyotime, Shanghai, China) for 5 min at room temperature and mounted with aqueous mounting medium. The results were imaged using a Nikon ECLIPSE 80i microscope.

### SH-SY5Y cells culture and treatment

SH-SY5Y cells were purchased from the Cell Storage Center of Wuhan University (Wuhan, China). SH-SY5Y cells were cultured in Dulbecco’s Modified Eagle Medium (DMEM, Invitrogen, Carlsbad, CA) supplemented with 10% fetal bovine serum (FBS, Invitrogen) and 1% antibiotics (100 units/ml penicillin, 100 μg/ml streptomycin). They were incubated in a humidified atmosphere containing 5% CO_2_ at 37 °C. Either glucose or mannitol was added as the high glucose (HG) group or the osmotic control, respectively (Fig. 1c). CCK8 assay was preformed to filtrate the optimum concentration of high glucose. Then, 1uM PERK inhibitor (GSK2656157, GSK) was choose to inhibit PERK activity of SH-SY5Y cells. Thus, the cells were divided into control group, HG group, HG + FGF1 group and HG + GSK.

### Statistical analyses

Data were presented as means ± SEM. Experiments were repeated at least 3 times, and the tissues from each replicate were from different mice. Using GraphPad Prism 5, statistical differences were determined by one-way analysis of variance (ANOVA) following Turkey test. Statistical significance was accepted when *p* < 0.05.

## Results

### Exogenous FGF1 ameliorates DICD with inferior learning and memory function

**Fig. 2:**
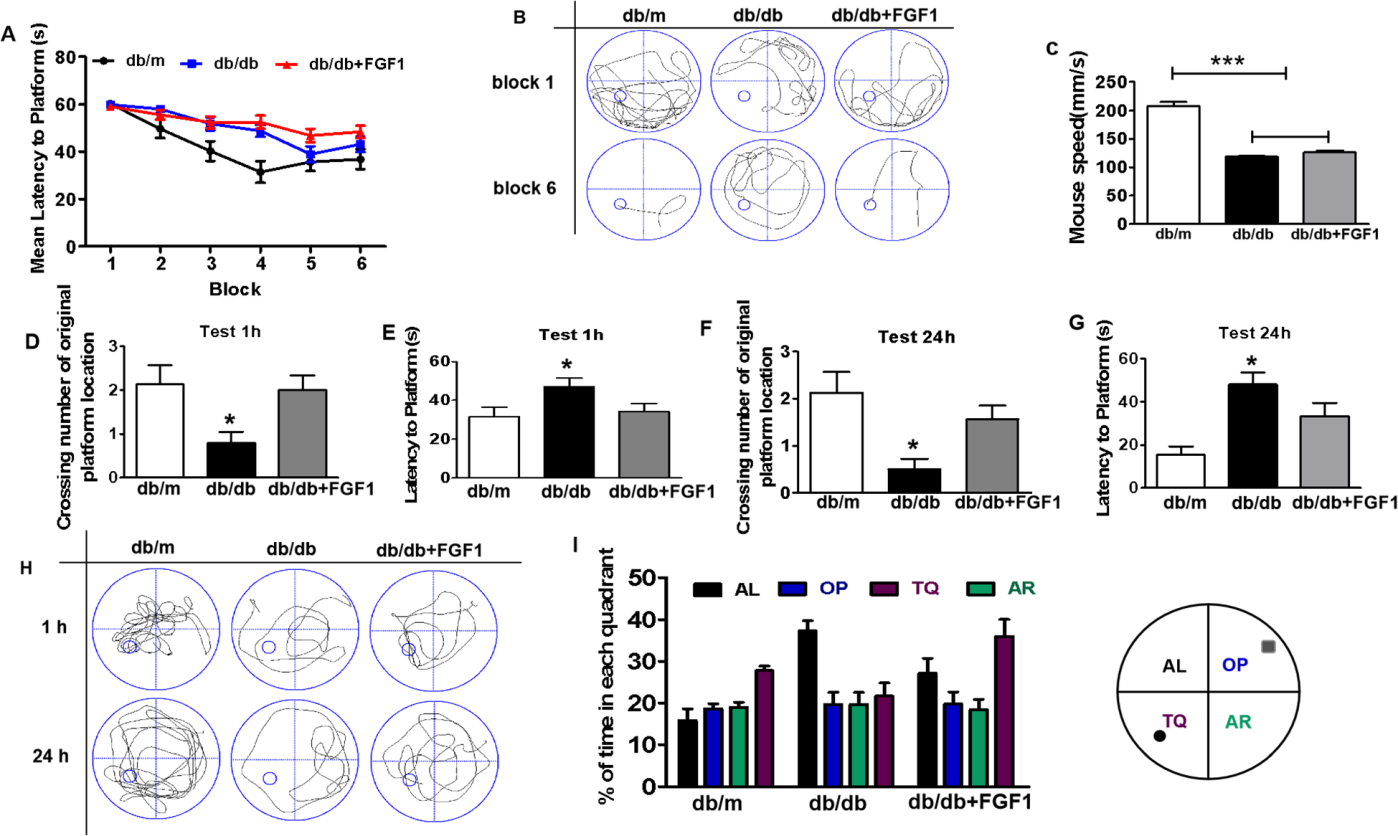
Exogenous FGF1 ameliorates DICD with inferior learning and memory function. **a** The learning curve of training period of mice during 6 blocks in the Morris water maze test; **b** Representative swimming track of mice at block 1 and block 6 during training period; **c** Swimming speed of mice in db/m, db/db and db/db + FGF1-treated mice during Morris water maze test; **d** Number of crossing over the original platform location of mice in probe trial (1 h after training); **e** Latency time to find the platform of mice in probe trial (1 h after training); **f** Number of crossing over the original platform location of mice in probe trial (24 h after training); **g** Latency time to find the platform of mice in probe trial (24 h after training); **h** Representative swimming track of mice in probe trial (1 h and 24 h after training); **i** Percentage of residence time in each quadrant of mice. The quadrant with platform was designated as TQ and the quadrant from which the mice started their swimming was designated as OP for “opposite”; The quadrant on the left side of OP was designated as AL for “adjacent left” and the quadrant on the right side of OP was designated as AR for “adjacent right”. *p < 0.05 vs. db/m mice and db/db + FGF1-treated mice, ***p < 0.001 vs. db/db mice and db/db + FGF1-treated mice, *n* = 10

All mice were trained to learn how to locate the platform throughout 6 blocks during training in Morris water maze test. As shown in Fig. 2a, db/db mice had to take longer than db/m mice to reach the platform from block 2 to block 6. After training, all the mice had significantly reduced the time to reach the platform (Fig. 2a), and FGF1-treated db/db mice shown shorter and more directional the swimming track to platform than those in db/db mice (Fig. 2b). Additionally, there was no significant difference of swimming speed between FGF1-treated db/db mice and db/db mice (Fig. 2c). Then, we had removed the platform at 1 h after training, and further tested the difference of spatial memory ability of mice on a probe trial. We found that db/db mice had fewer number of crossing over the platform position and took longer in latency to platform compared to those in db/m mice (Fig. 2d and e, *p* < 0.05). After 24 h, the storage memory of platform location was still worse in db/db mice during the probe trial, as indicated by fewer number of crossing over the platform (Fig. 2f and g, *p* < 0.05). Moreover, the swimming track of mice during the probe trial further indicated that db/db mice had worse memory than those in db/m mice (Fig. 2h). More importantly, exogenous FGF1 treatment had effectively ameliorated them (Fig. 2d-h, *p* < 0.05). Similar to those in db/m mice, FGF1-treated db/db mice had spent more time than db/db mice at quadrant of platform during trial (Fig. 2i). Taken together, these studies have suggested that FGF1 treatment effectively ameliorates diabetes-induced inferior spatial learning and memory function of mice.

### Role of FGF1-mediated regulation of glucose metabolism on DICD

**Fig. 3:**
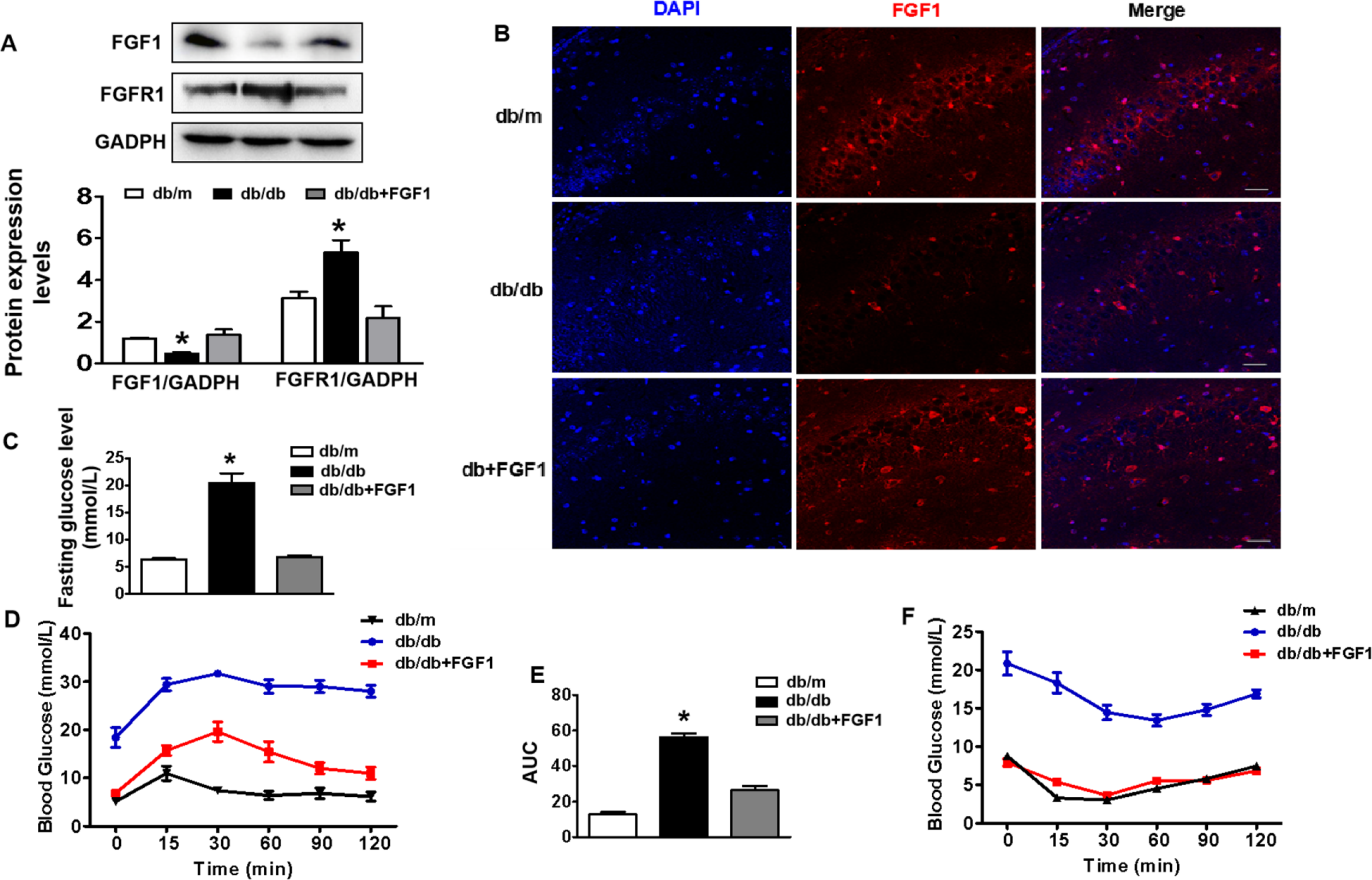
Role of FGF1-mediated glucose metabolism regulation on DICD. **a** Western blotting and quantitative analysis of FGF1 and FGFR1 expression in the hippocampus from db/m mice, db/db mice and db/db + FGF1-treated mice (*n* = 3); **b** The immunofluorescence staining result of FGF1 in the CA1 of hippocampus from mice (Scale bar = 15 μm), *n* = 3; **c** Fasting glucose level of mice (*n* = 10); **d** and **e** Blood glucose levels and AUC analysis of mice during GTT (*n* = 10); **f** Blood glucose levels of mice during ITT (*n* = 10); *p < 0.05 vs. db/m mice and db/db + FGF1-treated mice

Here, we had determined FGF1 expression in hippocampus under hyperglycaemia condition. It was observed that diabetes had significantly decreased FGF1 expression and enhanced FGFR1 expression in hippocampus when compared with those in db/m mice (Fig. 3a and b). Using GTT and ITT, we had further confirmed that FGF1 treatment had remarkably lowered fasting glucose level, remitted impaired glucose tolerance and insulin resistance (Fig. 3c-f). To verify whether normalizing hyperglycemia is the main contribute factor for FGF1 treating DICD, 200 mg/kg metformin, a classical anti-diabetic drug, was administrated to db/db mice as positive control group. It was observed that although both FGF1 and metformin administration effectively ameliorated metabolic indices of db/db mice (Additional file 1: Figure S1A and B), FGF1-treated db/db mice had showed better learning and memory function than those in metformin-treated db/db mice (Additional file 1: Figure S1C-F). These studies indicate that except for normalizing hyperglycemia function, there is another potential molecular mechanism underlying FGF1 ameliorating DICD.

### FGF1 blocks diabetes-induced morphological structure change and neuronal apoptosis in hippocampus during DICD

**Fig. 4:**
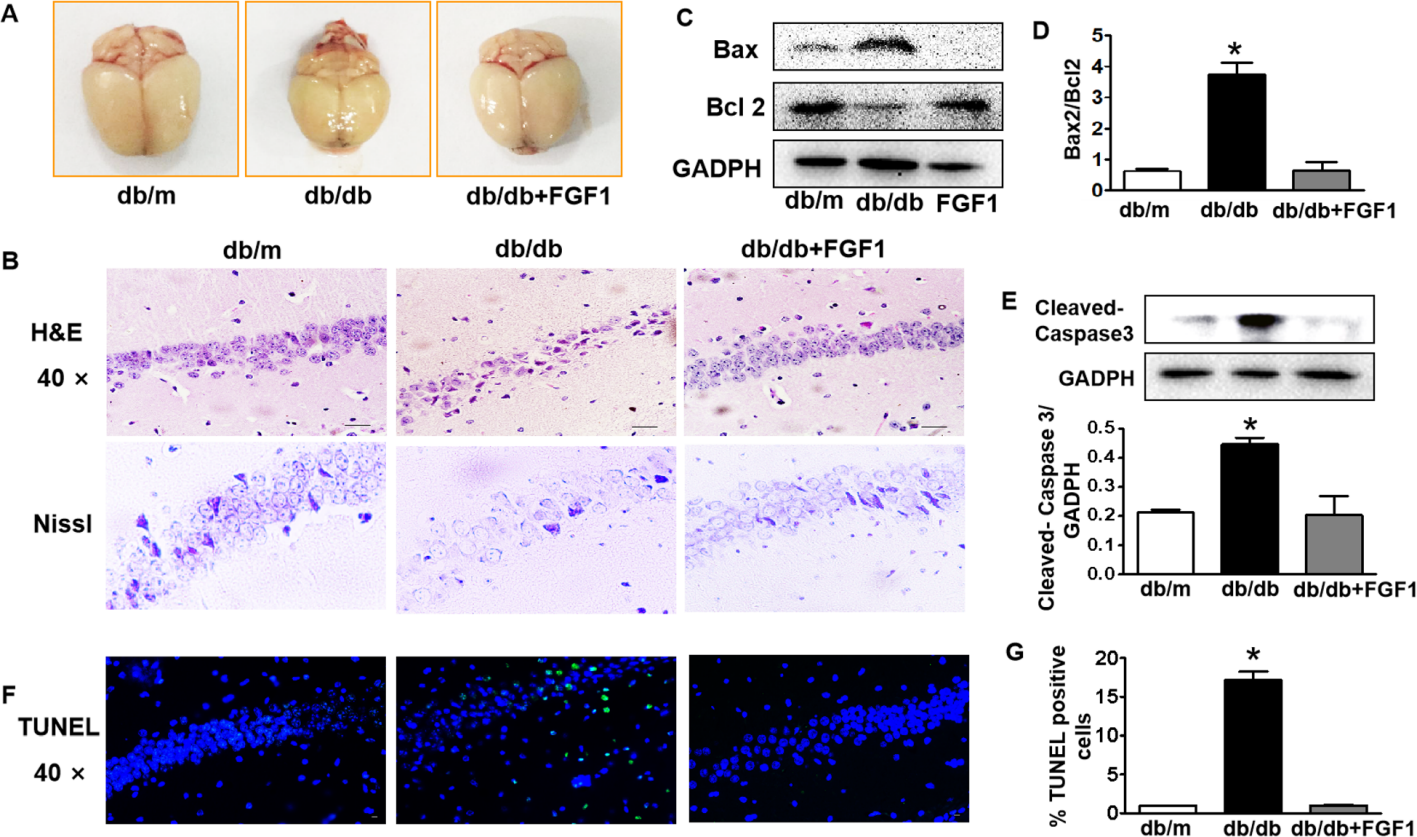
FGF1 blocks diabetes-induced morphological structure change and neuronal apoptosis in the hippocampus during DICD. **a** Morphological appearance of brains from db/m mice, db/db mice and db + FGF1-treated mice; **b** The H&E staining and Nissl staining of CA1 in the hippocampus from mice (Scale bar = 15 μm); **c-e** Western blotting and quantitative analysis of Bax, Bcl2 and Cleaved-Caspase3 expressions in the hippocampus from mice; **f** Representative images of TUNEL staining showing apoptotic cells (green signal) in the CA1 of hippocampus, cell nuclei were stained with DAPI (blue) (Scale bar = 15 μm); (**g**) The quantification of TUNEL-positive cells in the CA1 of hippocampus from mice. *p < 0.05, ***p < 0.001 vs. the other two groups, *n* = 3

Next, we had monitored morphological structure change and neuronal apoptosis of hippocampus in mice. We found that there is a certain amount of shrinkage of brain from db/db mice when comparing with that in FGF1 administration group (Fig. 4a). Additionally, the neuronal cells in the CA1 region of hippocampus from db/db mice exhibited an extensive loss, karyopyknosis, unclear cell membrane and sparse arrangement (Fig. 4b), while FGF1-treated db/db mice had normal neuronal cells structure with a large round nuclei and close arrangement (Fig. 4b). Furthermore, FGF1 administration significantly blocked diabetes-induced decrease of Bcl-2 expression and increase of Bax expression, subsequently upregulated Cleaved-Caspase3 expression (Fig. 4c-e, *p* < 0.05). Consistent with western blotting results, there is obvious much more TUNEL-positive cells in the hippocampus of db/db mice when compared to that in db/m mice, which was significantly reversed by FGF1 treatment (Fig. 4f and g, *p* < 0.05).

### Exogenous FGF1 alleviates diabetes-induced Aβ_1–42_ deposition and synaptic dysfunction

**Fig. 5:**
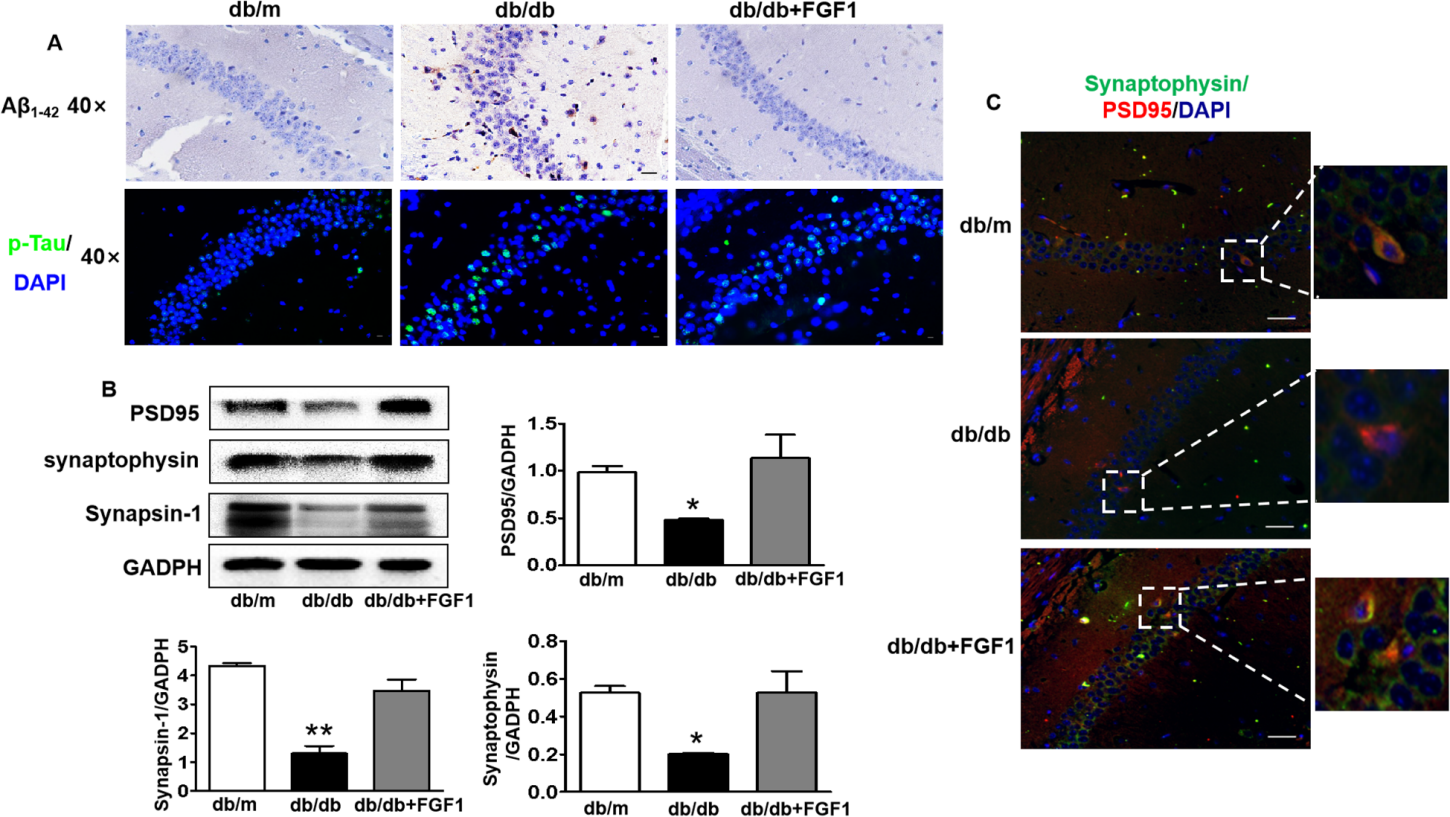
Exogenous FGF1 alleviates diabetes-induced Aβ_1–42_ deposition and synaptic dysfunction. **a** The immunohistochemical staining of Aβ_1–42_ and immunofluorescence staining of p-Tau in the CA1 of hippocampus (Scale bar = 15 μm); **b** Western blotting and quantitative analysis of synaptic function related protein expressions (PSD95, synaptophysin and synapsin-1) in the hippocampus; **c** Co-immunofluorescence staining of PSD95 and synaptophysin in the CA1 of hippocampus (Scale bar = 15 μm). *p < 0.05, **p < 0.01 vs. the other two groups, *n* = 3

Aβ_1–42_ deposition is the symbolic characteristics of AD. Here, we found that Aβ_1–42_ was largely deposited in the CA1 region of hippocampus from db/db mice when compared with that in db/m mice, which was partially reversed by FGF1 administration (Fig. 5a). Moreover, phosphorylated(p)-Tau level in the CA1 region of hippocampus from db/db mice was also remarkably increased than those in db/m mice and FGF1 administration group (Fig. 5a). Additionally, we had further explored the role of diabetes on synaptic function, and found that the expressions of synaptic function related proteins (PSD95, synaptophysin and synapsin-1) in hippocampus were remarkably suppressed under hyperglycemia, which were significantly reversed by FGF1 treatment (Fig. 5b). Moreover, we had further co-stained PSD95 (the scaffold proteins on postsynaptic membrane) and synaptophysin (the presynaptic membrane protein that usually used as a marker of synaptic density) to quantify the synapse number. The results had shown that PSD95 and synaptophysin were co-located in the neuron of hippocampus from db/m mice, but not in db/db mice (Fig. 5c). Exogenous FGF1 treatment had effectively reversed it (Fig. 5c). Taken together, FGF1 administration effectively alleviates diabetes-induced Aβ_1–42_ deposition and synaptic dysfunction.

### Exogenous FGF1 alleviates diabetes-induced elevated ER stress in the hippocampus during DICD

**Fig. 6:**
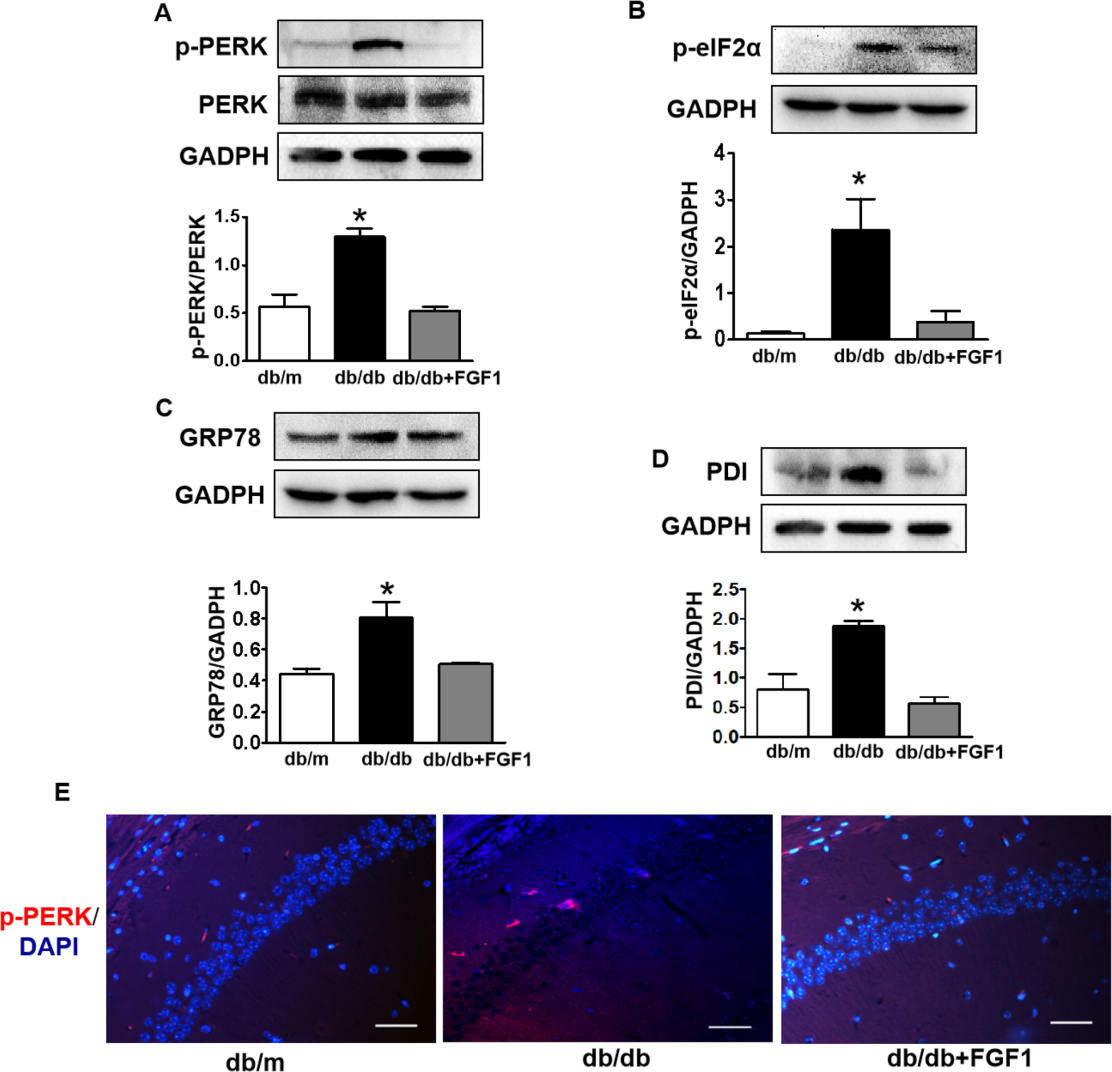
Exogenous FGF1 alleviates diabetes-induced elevated ER stress in the hippocampus during DICD. **a-d** Western blotting and quantitative analysis of p-PERK, p-eIF2α, GRP78, and PDI expressions in the hippocampus from db/m mice, db/db mice and db/db + FGF1-treated mice; **e** Immunofluorescence staining of p-PERK in the CA1 of hippocampus from mice (Scale bar = 15 μm). *p < 0.05 vs. the other two groups, *n* = 3

Then, we had also detected whether ER stress is involved in DICD development. It was observed that diabetes remarkably increased the expressions of p-PERK, phosphorylated eukaryotic initiation factor 2α (p-eIF2α), glucose regulated protein 78 (GRP78) and protein disulfide isomerase (PDI) in the hippocampus of mice when comparing with those in db/m mice (Fig.6a-d). FGF1 administration significantly suppressed them (Fig.6a-d). Consistent with western blotting results, FGF1 treatment significantly reversed diabetes-induced upregulation of the fluorescence intensity of p-PERK in the CA1 of hippocampus (Fig.6e). All of these results suggest that inhibition of PERK-eIF2α signaling maybe the vital molecular mechanism underlying FGF1 treating DICD.

### FGF1 alleviates diabetes-induced suppression of CREB activity and BDNF expression via PI3K-AKT signaling and PERK signaling

**Fig. 7:**
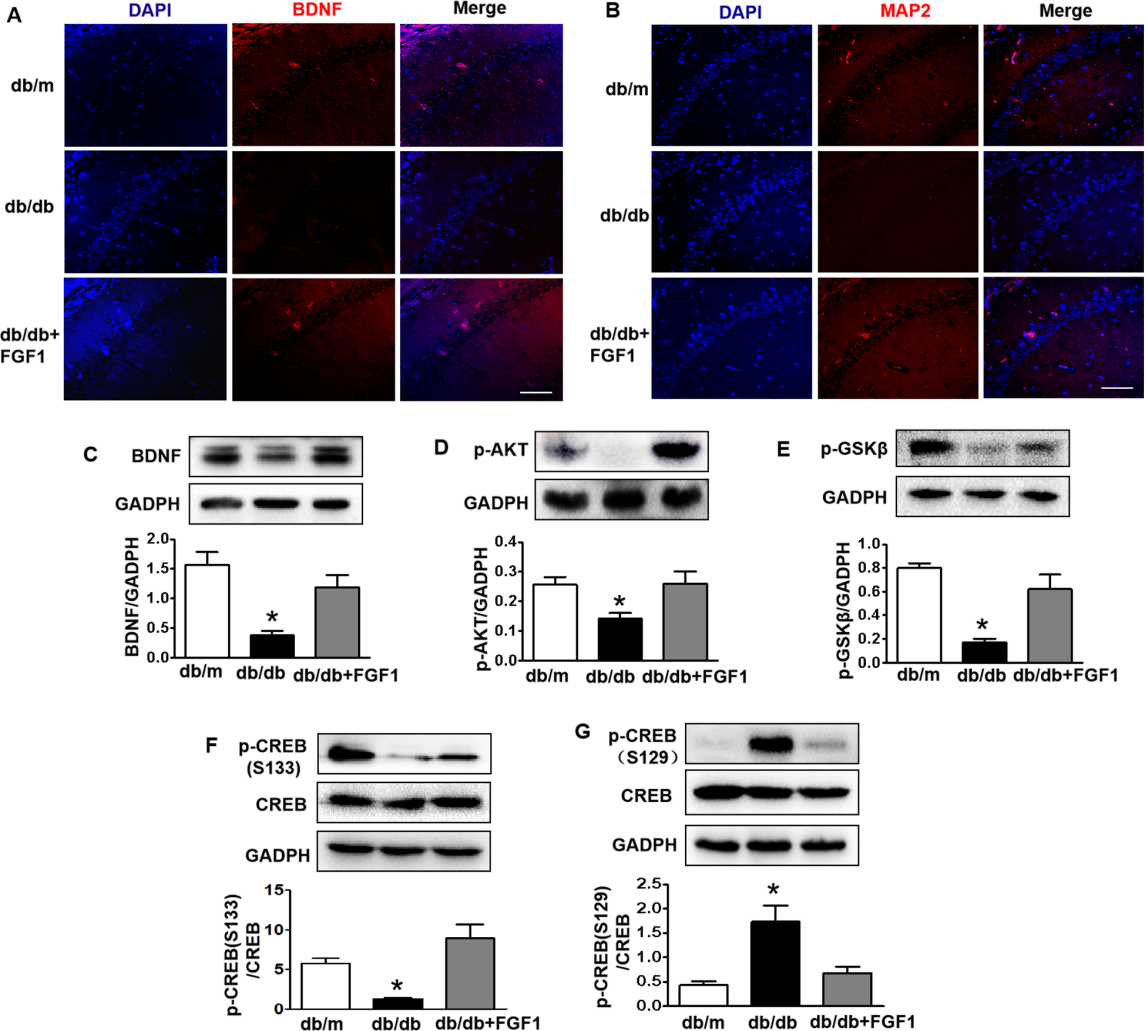
FGF1 alleviates diabetes-induced suppression of CREB activity and BDNF expression via PI3K-AKT signaling and PERK signaling during DICD. **a** and **b** Immunofluorescence staining of BDNF and MAP 2 in the CA1 of hippocampus from db/m mice, db/db mice and db/db + FGF1-treated mice (Scale bar = 15 μm); (**c**-**g**) Western blotting and quantitative analysis of BDNF, p-AKT, p-GSKβ, p-CREB (S133) and p-CREB (S129) expressions in the hippocampus from mice. *p < 0.05 vs. the other two groups, *n* = 3

In this study, we found that diabetes obviously suppressed the expressions of BDNF and MAP 2 in the hippocampus when comparing with those in db/m mice, and which were abolished by FGF1 administration (Fig.7a-c). CREB is a critical transcription factor that regulates BDNF and affects the formation and maintenance of memory [29]. We found that expression levels of p-CREB (S133), p-AKT and p-GSK3β in the hippocampus were significantly suppressed by diabetes (Fig.7d-f). FGF1 administration significantly blocked them (Fig.7d-f). CREB (S129) is one of downstream molecular of PERK, and p-CREB (S129) inhibits CREB activity. Here, we found that diabetes significantly enhanced p-CREB (S129) level in the hippocampus, which was reversed by FGF1 treatment (Fig.7g). These results suggest that FGF1 administration may alleviate diabetes-induced suppression of BDNF via coordinately enhancing AKT-GSK3β-CREB (S133) signaling and inhibiting PERK-CREB (S129) signaling in hippocampus during DICD.

### PERK inhibitor significantly suppresses PERK signaling and consequently blocks DICD

**Fig. 8:**
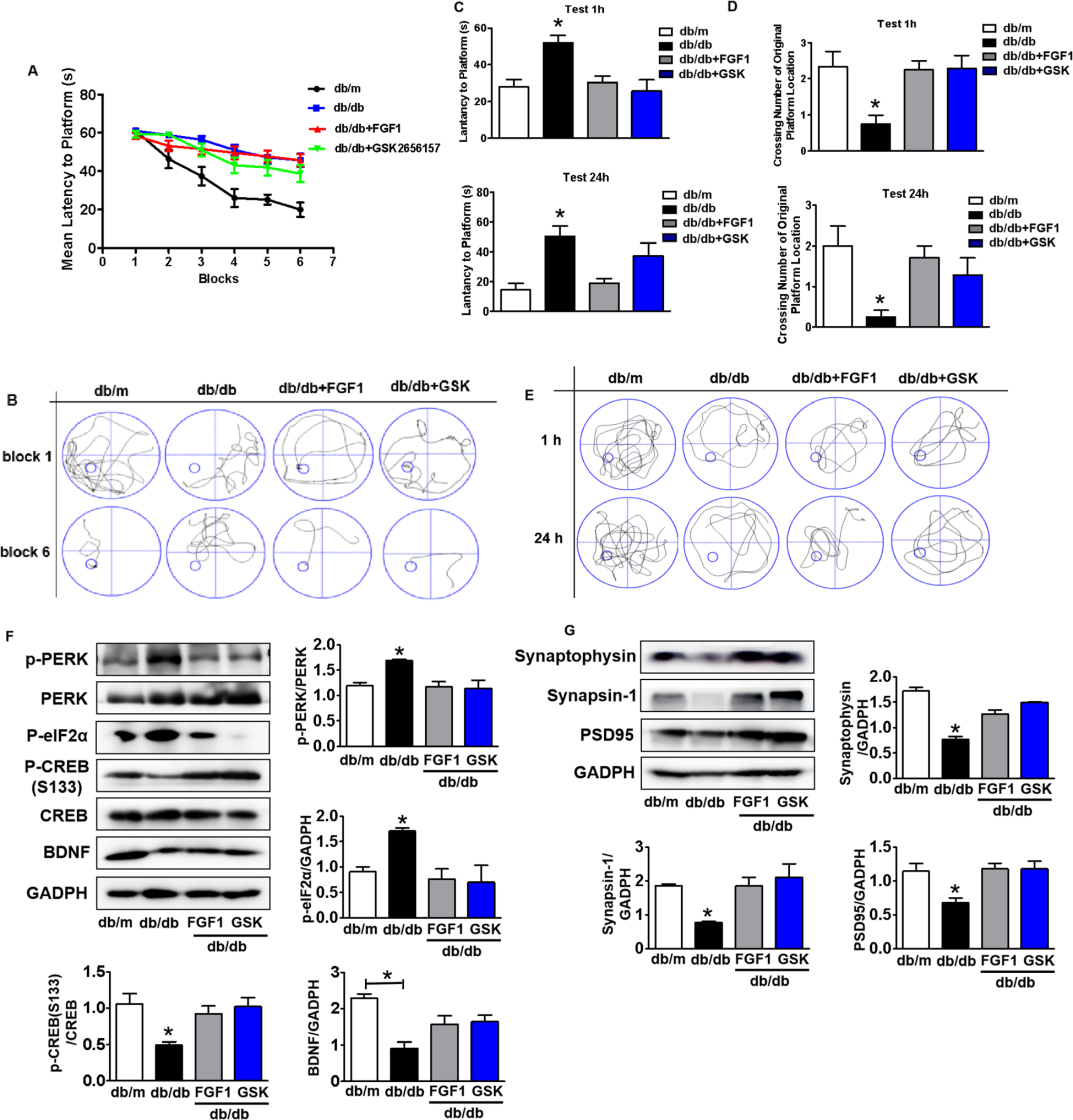
PERK inhibitor treatment significantly inhibits PERK signaling and consequently ameliorates DICD. **a** The learning curve of training period during 6 blocks in the Morris water maze test of mice after treating with GSK2356157 or FGF1, *n* = 8; **b** Representative swimming track of mice at block 1 and block 6 during training period; **c-d** Number of crossing over the original platform location and latency time to find the platform of mice in probe trial (1 h and 24 h after training) after treating with GSK2356157 or FGF1, *n* = 8; **e** Representative swimming track of mice in probe trial (1 h and 24 h after training); (**f**) Western blotting and quantitative analysis of p-PERK, p-eIF2α, BDNF and p-CREB (S133) expressions in the hippocampus of mice after treatment with GSK2356157 or FGF1, *n* = 3; **g** Western blotting and quantitative analysis of synaptic function related protein expressions (PSD95, synaptophysin and synapsin-1) in the hippocampus of mice after treatment with GSK2356157 or FGF1, *n* = 3. GSK: GSK2356157. *p < 0.05 vs. the other two groups

Then, we had used GSK2656157 (GSK), a PERK activity inhibitor, to treat mice and further detected the role of PERK signaling during FGF1 treating for DICD. Consistent with FGF1 administration, GSK treatment had a remarkably better cognitive function with superior spatial learning and memory function in db/db mice (Fig.8a-e). GSK treatment significantly inhibited PERK-eIF2α signaling evidencing with suppression of p-PERK and p-eIF2α expressions, and ameliorated suppression of p-CREB (S133) and BDNF expressions in the hippocampus of db/db mice (Fig.8f). Furthermore, it was observed that the diabetes- mediated suppression of PSD95, synaptophysin and synapsin-1 expressions in hippocampus were markedly reversed by GSK treatment, which is consistent with those in FGF1-treated group (Fig.8g). Taken together, these results indicate that PERK signaling exerts a pivotal role during FGF1 ameliorating DICD.

### PERK inhibitor blocks high glucose (HG)-induced neuronal apoptosis through inhibiting ER stress and enhancing CREB activity

Next, we had further investigated the neuroprotective role of FGF1 on neuron in vitro, and whether PERK signaling is involved in this process. Here, high glucose stimulating SH-SY5Y cells is used as neuronal model. SH-SY5Y cells that treated with 100 mM high glucose for 48 h exhibited significantly lower of cell viability than that in 50 mM high glucose condition (Additional file 1: Figure S2A and B). Moreover, the expressions of ER stress-related proteins and Cleaved-Caspase3 were remarkably induced in SH-SY5Y cells under 100 mM high glucose for 48 h (Additional file 1: Figure S2C-E). Thus, SH-SY5Y cells cultured with 100 mM glucose for 48 h were considered as HG group.

**Fig. 9:**
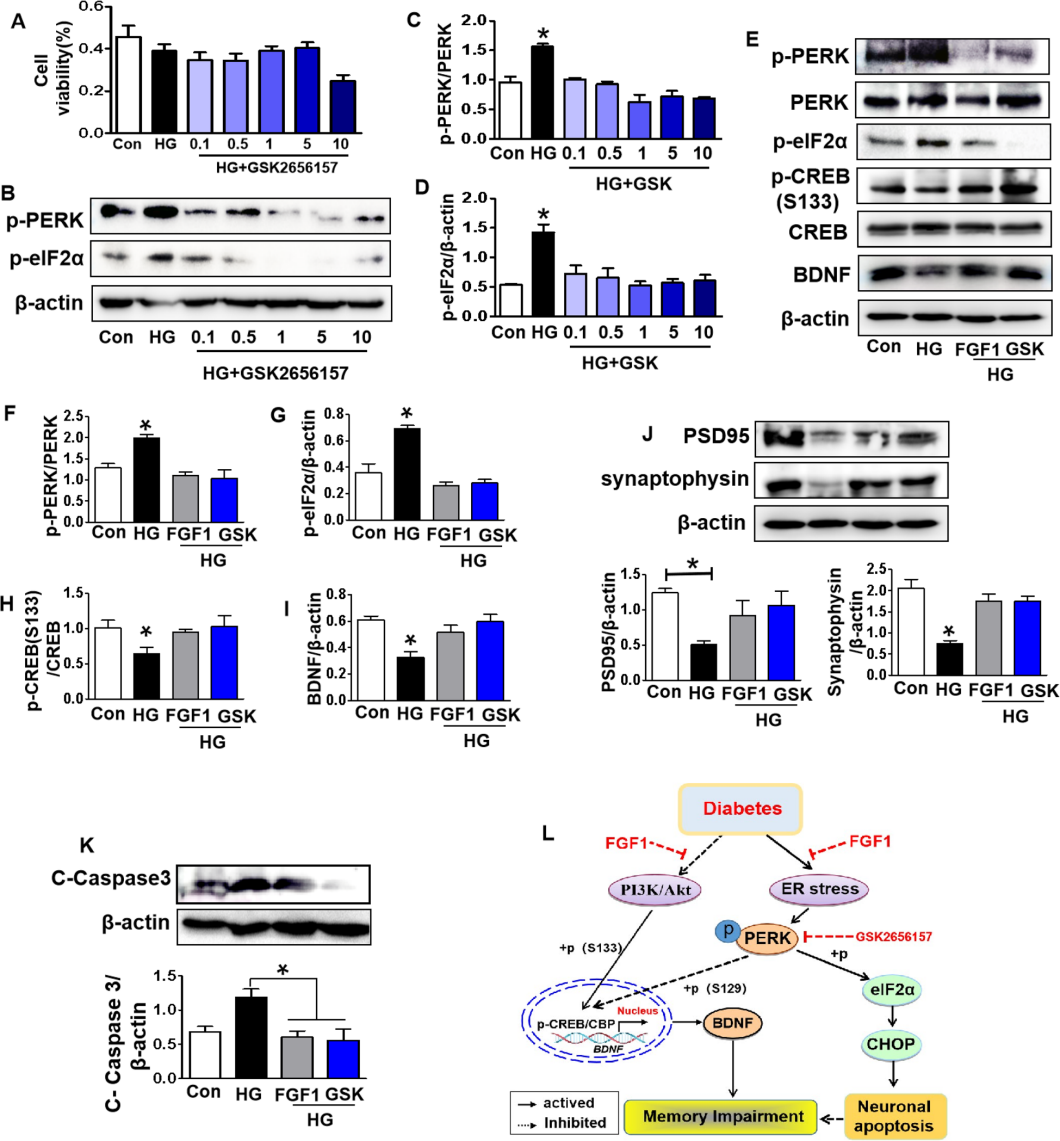
PERK inhibitor blocks high glucose-induced neuronal apoptosis through inhibiting ER stress and enhancing CREB activity. **a** Cell viability of SH-SY5Y cells under high glucose with different concentrations of GSK2356157; **b-d** Western blotting and quantitative analysis of p-PERK and p-eIF2α expressions in SH-SY5Y cells under high glucose with or without different concentrations of GSK2356157; **e-k** Western blotting and quantitative analysis of p-PERK, p-eIF2α, BDNF, p-CREB (S133), PSD95, synaptophysin and Cleaved-Caspase 3 expressions in SH-SY5Y cells under high glucose treating with GSK or FGF1. GSK: GSK2356157. *p < 0.05 vs. the other two groups, *n* = 3. **j** A Schematic showing FGF1 ameliorates diabetes-induced cognitive dysfunction

Then, GSK was used to treat SH-SY5Y cells under HG condition. We found that the SH-SY5Y cells treated with 1 uM GSK had a better cell viability, and PERK-eIF2α signaling was inhibited in SH-SY5Y cells under HG + GSK condition (Fig.9a-d). FGF1 treatment significantly reversed HG-induced upregulation of p-PERK, p-eIF2α, and Cleaved-Caspase3, and blocked the suppression of p-CREB (S133), BDNF, PSD95 and synaptophysin in SH-SY5Y cells (Fig.9e-k). Moreover, there were no significant differences in the expressions of these proteins in SH-SY5Y cells between HG + GSK group and HG + FGF1 group (Fig.9e-k).

## Discussion

Diabetes causes central nervous system damage, resulting in the structural and functional changes, and cognitive decline [30]. In the current study, we used db/db mice as DICD animal model, and confirmed that diabetes remarkably causes cognitive decline with inferior spatial learning and memory function. Furthermore, Aβ_1–42_ was largely deposited in the hippocampus of db/db mice, and synaptic dysfunction in the hippocampus was presented in db/db mice. These findings are consistent with our and others’ previous studies [1, 31]. With the increasing epidemic of diabetes, it is urgent need to deeply elucidate the mechanisms underlying DICD, and investigate the reasonable and effective treatment strategies for it.

FGF1 has dual function of normalizing hyperglycemia [23] and neuroprotection [32]. In this study, diabetes significantly reduced FGF1 expression in the CA1 of hippocampus, suggesting that FGF1 may be an key regulatory factor during DICD development. Based on the dual function of FGF1 and its expression level in the hippocampus, exogenous FGF1 was choose to treat DICD. It confirmed our hypothesis that FGF1 treatment effectively improves diabetes-induced inferior spatial learning and memory function.

Although type 2 diabetes is a complex metabolic disorder, hyperglycemia with resulting glucotoxicity is a major mediator of diabetes-induced complication. In the current study, FGF1 administration reduced hyperglycemia, suggesting that hyperglycemia may be also the key caused factor for DICD occurrence and development. But FGF1-mediated normalizing hyperglycemia may not the only contributed factor for FGF1 treating DICD, which may explain why FGF1 treatment exerts a better neuroprotective role for DICD when comparing with that in metformin treatment group. Moreover, due to its large molecular size of 17 kDa, the 154-amino acid protein FGF1 is unable to freely pass through biological membranes and the blood-brain barrier (BBB) [33]. However, increased blood-brain barrier (BBB) permeability is a critical neurovascular complication of T2DM that adversely affects the central nervous system homeostasis and function [34]. Thus, it is reasonably speculated that independent of normalizing hyperglycemia, FGF1 may directly exert its neuroprotective role and improve the cognitive function after permeating BBB, which need to be further confirmed in further study.

As shown in Fig. 2b, the motor function of db/db mice is significantly decreased when compared with the db/m group. It can be speculated that obesity may affect their motor speed, or long-term hyperglycemia may cause the impairment of their motor function. In our current study, however, FGF1 treatment did not increase its motor speed of db/db mice, suggesting that hyperglycemia did not affect motor function. Of course, it should be further study.

Excessive ER stress and subsequent caspase-dependent apoptosis are the major causal events for diabetes-induced complications [27, 35, 36]. During the early stage of embryonic development, maternal hyperglycemia activates PEKR-eIF2α signaling pathway, resulting in the neural tube defects [27, 35]. In this study, diabetes significantly triggered PERK-eIF2α signaling and induced excessive apoptosis in the hippocampus, and FGF1 treatment blocked them. These results further suggest that chronic hyperglycemia is a key caused factor for DICD development, and normalizing hyperglycemia is a critical molecular mechanism underlying FGF1 treatment for DICD. Additionally, we found that independent of eIF2α, p-PERK inhibits CREB activity by increasing p-CREB (S129), which is consistent with prior study in TBI animal model [14]. These findings indicate that PERK signaling is a major regulatory pathway during DICD development, and which is further confirmed in vivo and in vitro by using PERK inhibitor with much better cognitive function of db/db mice and SH-SY5Y cells survival under HG condition.

BDNF, a neurotrophic factor, is widely expressed in the CNS and involved in the regulation of neurite growth [37], directional guidance, induction of LTP and release of neurotransmitters [38]. BDNF deficiency is closely associated with cognitive decline [7, 39]. In the current study, diabetes significantly suppressed BDNF expression and p-CREB (S133) levels in the hippocampus, and PERK inhibitor had improved the BDNF expression and CREB activity, which were consistent with those in FGF1 treatment group. Taken together, these studies reveal that inhibition of CREB-BDNF signaling is essential for DICD development, and PERK signaling is involved in this process.

AKT is the key kinase for the regulation of CREB activity [40–42]. In our current study, diabetes remarkably inhibited PI3K/AKT signaling and reduced p-CREB (S133) level in the hippocampus, which was significantly blocked by FGF1 administration. All of these results suggest that coordinating with PERK signaling, PI3K/AKT signaling is involved in the regulation of CREB activity during DICD. However, it is still unclear whether diabetes directly regulates PI3K/AKT signaling. Prior studies have demonstrated that miRNAs are involved in the regulation of cognitive function [43–46], such as miR-206, miR-107, miR-328, and miR-29. Series of studies have shown that miR-206 is highly expressed in the brain of AD patients or AD animal models, which contribute to cognitive decline by inhibiting BDNF expression [47, 48]. Additionally, miR-206 can regulate IGF-1 expression, and activate PI3K/AKT signaling [49, 50]. Thus, we speculate that miR-206 could regulate IGF-1 expression, affect PI3K/AKT/CREB signaling during DICD. This need to be further detected.

## Conclusions

In summary, diabetes significantly induces cognitive dysfunction. Mechanistic studies demonstrated that diabetes significantly induces PERK signaling in hippocampus, which not only triggers neuronal apoptosis, but also reduces CREB activity by activating p-CREB (S129) (Fig. 9l). Coordinating with PERK signaling, diabetes remarkably inhibits PI3K/AKT signaling and suppresses p-CREB (S133) level, subsequently inhibits CREB activity (Fig. 9l). FGF1 effectively blocks diabetes-induced neuronal apoptosis and BDNF reduction, consequently ameliorates DICD though inhibiting PERK signaling and promoting PI3K/AKT signaling (Fig. 9l). These studies suggest that FGF1 maybe a effective and potential strategy for clinically treating DICD, which may lead to a novel therapeutic intervention for DICD.

## Supplementary information

**Additional file 1: Figure S1.** Metformin treatment ameliorates diabetes-induced cognitive decline with inferior learning and memory function. **Figure S2.** The effect of high glucose on neuronal cell apoptosis and ER stress in vitro.

## Data Availability

The datasets used and/or analysed during the current study are available from the corresponding author on reasonable request.

## Abbreviations

DICD: Diabetes-induced cognitive decline
FGF1: Fibroblast growth factor 1
CREB: cAMP-response element binding protein
CBP: CREB binding protein
BDNF: Brain derived neurotrophic factor
PKA: protein kinase A
AKT: protein kinase B
AD: Alzheimer disease
ER: Endoplasmic reticulum
PERK: Protein kinase RNA-like ER kinase
eIF2α: eukaryotic initiation factor 2α
GRP78: Glucose regulated protein 78
PDI: Protein disulfide isomerase
TBI: Traumatic brain injury
PD: Parkinson’s disease
Met: Metformin
GSK: GSK2656157
HG: High glucose
BBB: Blood-brain barrier
