# Supplementary figures and images for: Exogenous fibroblast growth factor 1 ameliorates diabetes-induced cognitive decline via coordinately regulating PI3K/AKT signaling and PERK signaling
Source: Cell Commun Signal. 2020 May 27;18:81. doi: 10.1186/s12964-020-00588-9 (PMC7251863; doi:10.1186/s12964-020-00588-9)

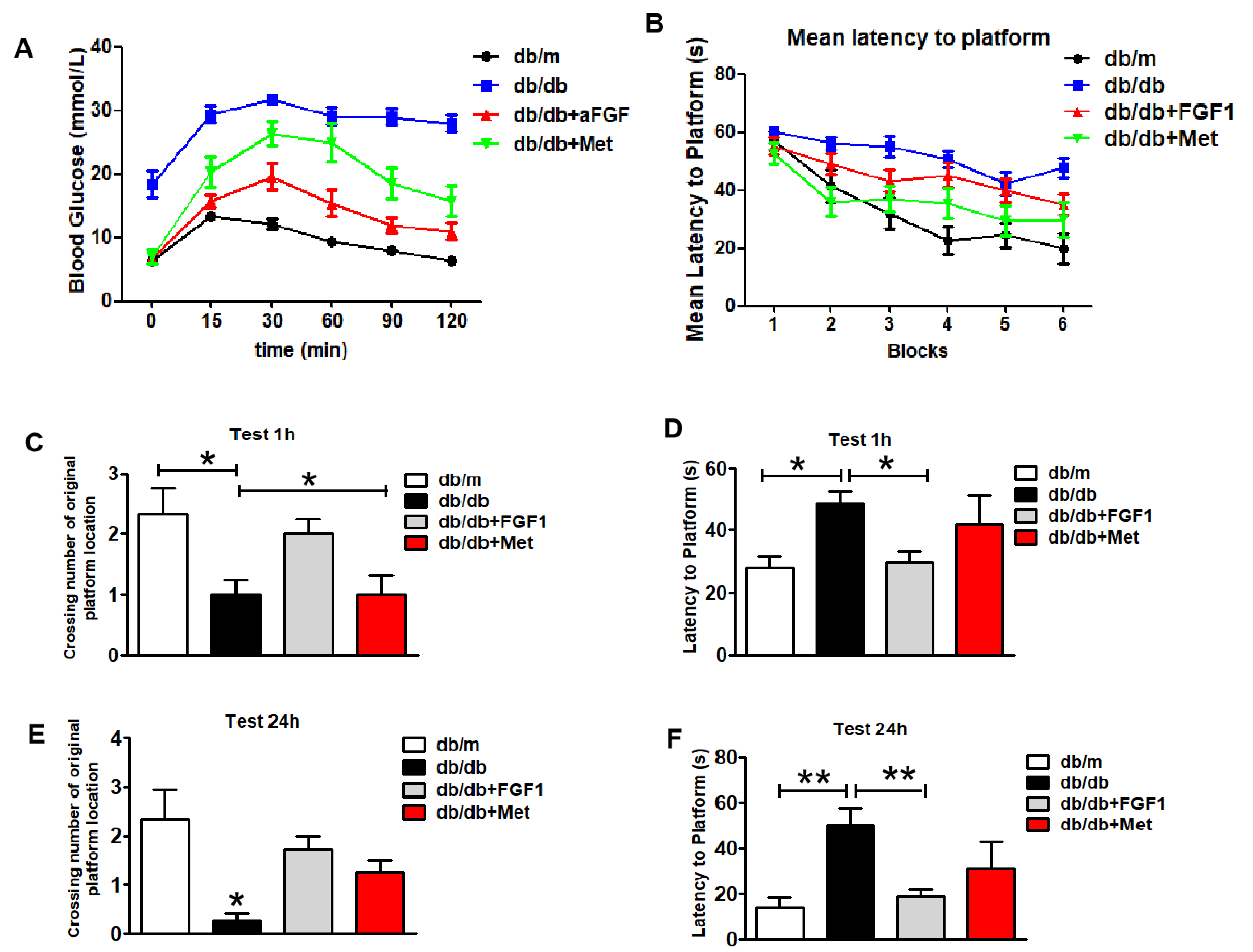

Supplement: Supplementary file 2 — Additional file 1: Figure S1. Metformin treatment ameliorates diabetes-induced cognitive decline with inferior learning and memory function. Figure S2. The effect of high glucose on neuronal cell apoptosis and ER stress in vitro. [file 12964_2020_588_MOESM2_ESM.zip › Supplementary Figure 1.tif]

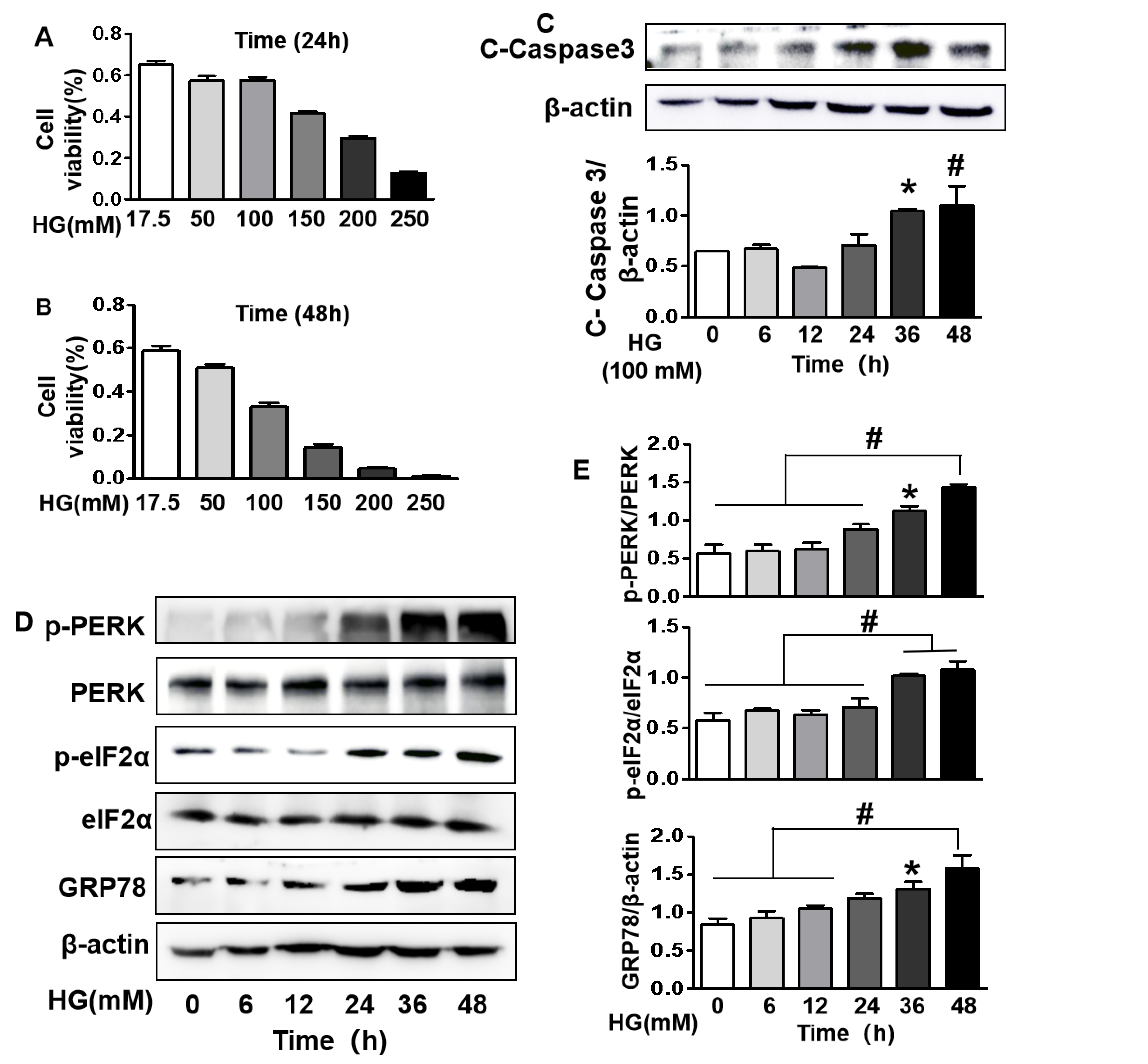

Supplement: Supplementary file 2 — Additional file 1: Figure S1. Metformin treatment ameliorates diabetes-induced cognitive decline with inferior learning and memory function. Figure S2. The effect of high glucose on neuronal cell apoptosis and ER stress in vitro. [file 12964_2020_588_MOESM2_ESM.zip › Supplementary Figure 2.tif]
