# Supplementary material for: Exogenous fibroblast growth factor 1 ameliorates diabetes-induced cognitive decline via coordinately regulating PI3K/AKT signaling and PERK signaling
Source: Cell Commun Signal. 2020 May 27;18:81. doi: 10.1186/s12964-020-00588-9 (PMC7251863; doi:10.1186/s12964-020-00588-9)
Supplement: Supplementary file 2 — Additional file 1: Figure S1. Metformin treatment ameliorates diabetes-induced cognitive decline with inferior learning and memory function. Figure S2. The effect of high glucose on neuronal cell apoptosis and ER stress in vitro. [file 12964_2020_588_MOESM2_ESM.zip › 2020-1-13 Supplementary Figure legends.doc]

**Supplementary figure legends**

**Supplementary Figure 1. Metformin treatment ameliorates diabetes-induced cognitive decline with inferior learning and memory function.** (A) Blood glucose levels of mice during GTT after treating with metformin or FGF1; (B) The learning curve of training period during 6 blocks in the Morris water maze test of mice after treating with metformin or FGF1; (C) Number of crossing over the original platform location of mice in probe trial (1 hr after training) after treating with metformin or FGF1; (D) Latency time to find the platform of mice in probe trial (1 hr after training) after treating with metformin or FGF1; (E) Number of crossing over the original platform location of mice in probe trial (24 hr after training) after treating with metformin or FGF1; (F) Latency time to find the platform of mice in probe trial (24 hr after training) after treating with metformin or FGF1. *p < 0.05, **p < 0.01 *vs.* the other groups, n(db/m)=7，n(db/db)=11, n(db/db+FGF1)=12, n(db/db+Met)=6.

**Supplementary Figure 2. The effect of high glucose on neuronal cell apoptosis and ER stress in vitro.** (A and B) Cell viability of SH-SY5Y cells treating with different concentrations of high glucose for 24h or 48h. (C-E) Western blotting and quantitative analysis of p-PERK, p-eIF2α, GRP78, and Cleaved-Caspase 3 expressions in SH-SY5Y cells treating with 100mM high glucose for different time points.
